# A hyperendemic focus of *Taenia solium* transmission in the Banke District of Nepal

**DOI:** 10.1016/j.actatropica.2017.07.022

**Published:** 2017-12

**Authors:** Keshav Sah, Ishab Poudel, Suyog Subedi, Dinesh Kumar Singh, Jo Cocker, Peetambar Kushwaha, Angela Colston, Meritxell Donadeu, Marshall W. Lightowlers

**Affiliations:** a Heifer International, G.P.O. Box 6043, Kathmandu, Nepal; b Department of Pathology and Clinics (HOD), Tribhuvan University, Institute of Agriculture and Animal Science, Rampur Campus, Chitwan, Nepal; c GALVmed (Global Alliance for Livestock Veterinary Medicines), Doherty Building, Pentlands Science Park, Bush Loan, Edinburgh EH26 0PZ, Scotland, UK; d GALVmed, Unit 118 & 120 B, Splendor Forum, Plot No 3, Jasola District Centre, Jasola, New Delhi 110025 India; e GALVmed, Galana Plaza, 4th Floor Wing C Suite B, Galana Road, Kilimani, Nairobi 00100 Kenya; f Veterinary Clinical Centre, Faculty of Veterinary and Agricultural Sciences, University of Melbourne, 250 Princes Highway, Werribee, Victoria 3030, Australia; g Initiative for Neglected Animal Diseases (INAND), Whitby Manor Office Estate, 14th Road, Midrand, South Africa

**Keywords:** *Taenia solium*, Cysticercosis, Pig, Nepal, Banke, Necropsy

## Abstract

Neurocysticercosis is a major cause of epilepsy in countries where *Taenia solium* is endemic and the parasite is a major cause of food-borne disease globally. Pigs are the natural intermediate host involved in transmission of the parasite. *T. solium* is known to be endemic in Nepal, however there is limited reliable data about the prevalence of the disease in Nepal. The aim of this study was to determine accurately the prevalence of porcine cysticercosis in slaughter age pigs in an area of Nepal where pigs are known to be free-roaming. Pigs were obtained from the Udaypur Village Development Committee (VDC) and Hirminiya & Betahani VDC of the Banke district in Nepal. One hundred and ten animals of slaughter age (approximately 8–16 months old) were purchased, slaughtered and the heart, liver, brain and half the body skeletal musculature were sliced using hand knives and the number and viability of *T. solium* cysts determined. Thirty two of the 110 animals were found to harbour *T. solium* cysticerci (29%), of which 30 (27%) were found to have viable cysticerci (93% of the infected animals). This is one of the highest prevalences of porcine cysticercosis that has been reported to date from the results of necropsy on randomly selected animals. This study highlights a high rate of transmission of *T. solium* in the Banke District of Nepal. It encourages further investigation of human and porcine cysticercosis in Nepal, as well as implementation of efforts to reduce transmission of the parasite and the associated human disease.

## Highlights

• The prevalence of porcine cysticercosis was determined in pigs from the Banke District of Nepal.
• Slaughter-age animals were selected at random and underwent detailed necropsy examination.
• Thirty two of 110 animals were found to harbour *T. solium* cysticerci.
• This is one of the highest levels of porcine cysticercosis described for any region in the world.
• Measures are warranted to control this zoonotic disease in Nepal.

## Graphical abstract

**Figure:**
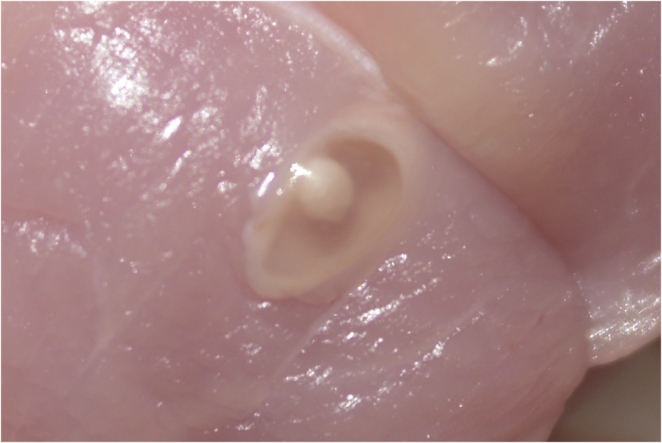

## 1 Introduction

*Taenia solium* is a zoonotic cestode parasite which causes cysticercosis in humans. Cysticercosis is a major cause of epilepsy in many developing countries; Ndimubanzi et al. (2010) identified 29% of seizure cases to be associated with neurocysticercosis in *T. solium* endemic regions. The disease is formally recognised by the WHO as a Neglected Tropical Disease (WHO, 2010), ranked as the most important food-borne parasitic infection from a global perspective by the Food and Agriculture Organization of the United Nations (Robertson et al., 2013) and accounts for the largest proportion of the global burden of disease caused by foodborne parasites (Havelaar et al., 2015).

*T. solium* is transmitted in a life cycle between humans, which act as the obligate definitive host, and pigs which act as the intermediate host. Full transmission of the life cycle of *T. solium* occurs in poor countries where pigs roam free and where meat hygiene and cultural practices favour the ingestion of poorly cooked pig meat harbouring the larval cysts.

Human neurocysticercosis is a frequently diagnosed condition in Nepal (Devleesschauwer et al., 2012, Joshi et al., 2007, Joshi et al., 2004, Joshi et al., 2001), where it had been estimated to be responsible for the highest burden of disease caused by a parasitic zoonosis (Devleesschauwer et al., 2014). Porcine cysticercosis is known to occur in Nepal (Joshi et al., 2007, Joshi et al., 2001) however relatively little information is available about the prevalence of *T. solium* infection in pigs. Devleesschauwer et al. (2013b) undertook a serological survey of porcine cysticercosis in pigs slaughtered in the Kathmandu valley, finding an infection rate of up to 28%. Maharjan and Gaihre (2010) performed serology on samples from pigs in the Syangja District of the Western Development Region in Nepal and found 23.5% to be positive. The antigen ELISA and EITB serological methods that were used in these studies are now understood to have a higher level of false positive reactions in pigs from *T. solium* endemic areas (Devleesschauwer et al., 2013a, Gavidia et al., 2013, Jayashi et al., 2014, Lightowlers et al., 2016) than had previously been understood, hence the reliability of these data is unclear. The only reliable and specific method currently available for determining the prevalence of porcine cysticercosis is a detailed post mortem carcase dissection that detects cysticerci in the tissues (Lightowlers et al., 2016).

The Banke district is one of 75 districts in Nepal, located in the mid-western part of the country bordering Indian Uttar Pradesh. It is a largely rural district with 85% of its approximately 0.5 million population living in rural villages (Ministry of Agriculture and Development, Nepal, 2016). Pigs are maintained under traditional husbandry conditions by the Khatik communities, where the animals are allowed to roam freely during the daylight hours and containment in a conventional enclosure at night time. Toilets are generally not present in the communities or, where they are present, they are often not used. Pigs may be kept in stys constructed using mud and bricks, the roof usually being straw. There is limited ventilation in these stys, making conditions inside damp and humid. The animals are often left to scavenge through the country side and the suburbs, but they may also be fed. The primary sources of water are boreholes and sanitation is neglected. Farmers do not use any anthelmintic for their animals. There is a paucity of awareness regarding taeniasis/cysticercosis among these communities. Commonly, the pigs are slaughtered on-farm where they were reared and the pork, without any inspection, enters into the human food chain, through local markets. Animals are also sold to slaughterhouses in regional centres where meat inspection may identify and condemn carcases heavily infected with *T. solium*.

In order to determine accurately the prevalence of porcine cysticercosis in a region of Nepal where *T. solium* transmission was thought likely to occur, a sample of pigs from the Banke district was investigated by detailed necropsy examination of selected organs and half the carcase musculature. The investigation was undertaken as part of a base-line study examining the effectiveness of a vaccination and chemotherapy intervention for porcine cysticercosis.

## 2 Materials and methods

### 2.1 Study design

A baseline survey was conducted in 184 pig rearing households in Udaypur Village Development Committee (VDC) and Hirminiya & Betahani VDC of the Banke district in Nepal (81°37′E–81°42′E, 27°90′N–28°20′N). These regions were selected because they were known to contain Dalit communities having many free-roaming pigs. The GPS coordinates of households were recorded together with household particulars, and a questionnaire completed concerning use of latrines or toilets, pig management and care, and knowledge and awareness of taeniosis and neurocysticercosis in humans and of cysticercosis in pigs. A randomized list of 110 household was obtained and one slaughter weight pig from each household was purchased in order to undertake post mortem examination. Typically, the households had only a single slaughter-weight animal that was available for purchase. The animals selected for post mortem may have been confined for part of the day, or of the year, but were not housed or confined for their entire life span. These included 55 animals from Udaipur, 31 animals from Hirmaniya and 24 animals from Betahani. The majority of the pigs were indigenous breeds. The age of the animals was recorded based on advice from the animal owners. The animal weight was estimated by experienced staff.

### 2.2 Post mortem procedures

The animals were transported to a licensed commercial abattoir in Nepalgunj Municipality, Banke where they were euthanized by slaughter house staff according to normal commercial practices. The viscera were removed and the heart, liver, both kidneys and the full diaphragm retained in numbered containers. The carcase was divided cranio-caudally. The organs and the right hand half of the carcase, including the complete head, were refrigerated overnight at 4 °C, after which the carcase was skinned. The head was removed and the tongue, masticatory muscles (both right and left sides) and brain removed and retained. The muscles from the right hand side of the carcase were dissected from the bones, keeping separate the muscles of the forelimb.

### 2.3 Examination for *Taenia solium* cysts

Except in cases of very heavy infection, all the retained organs and muscles were sliced by hand at intervals of approximately 3 mm and examined meticulously for the presence of *T. solium* cysticerci or other lesions. Cysticerci were recorded as viable where they were translucent vesicles filled with transparent fluid and having a visible white scolex. Non-viable lesions were recorded separately in cases where vesicles were non-translucent, containing a dense white or yellowish fluid and having no scolex and in cases of fibrosed or calcified lesions. In cases where it was clear that a carcase contained thousands of cysts, all of the heart, liver, kidneys, diaphragm, tongue, masticatory muscles, forelimb and brain were sliced and counted as above. The remaining carcase musculature was weighed and representative samples from different muscle sites were selected representing approximately 1 kg. This sample was weighed accurately and then sliced and counted as above.

### 2.4 Estimation of the burden of *Taenia solium* in each carcase

For carcases where all the musculature from the right half of the carcase was sliced, the numbers of cysticerci in the whole carcase were estimated by doubling the number recorded in the carcase half that was sliced, and adding the number recorded for the full diaphragm, tongue, masticatory muscles, heart, liver, kidneys and brain. For carcases having very heavy levels of infection, the total carcase burden was estimated by adding the numbers for the diaphragm, tongue, masticatory muscles, heart, liver, kidneys, and brain plus twice the number found in the foreleg, plus the numbers found in the 1 kg sample multiplied by the total weight of carcase musculature that was sampled.

### 2.5 Definition of a case of confirmed porcine cysticercosis

An animal was determined to be a confirmed case of porcine cysticercosis if one or more viable cysticerci were found in the muscle and or the brain, or if more than one non-viable lesion was detected in the muscles and/or brain.

## 3 Results

### 3.1 Survey

The survey of 110 households found that they held a total of 578 pigs. The majority of households (80.9%) did not have access to latrine and 95% of the households confirmed that their pigs had access to human faeces. Most of the pigs (92.7%) were of indigenous breeds and 7.3% were crossbreed. Most pigs were free ranging and 89% penned the animals at night, 2.7% allowed their animals to be permanently free ranging, 2.7% tethered the pigs and 5.5% of the animals were reared intensively. The major reason for rearing pigs was for sale to the local market (85.5%) with 13.6% being for both home consumption and sale. An awareness regarding tapeworm infection was found in 34.5% of the households and 16.4% recognised a relationship between eating raw/undercook pork and being infected with tapeworm. Almost all households (91.8%) affirmed that they had found cysts in pig meat during meat preparation. Most households (88.2%) were aware of epilepsy, headache and skin nodules and 16% were able to relate tapeworm infection with symptoms such as epilepsy, headache and skin nodules.

### 3.2 Post mortem examination

**Fig. 1:**
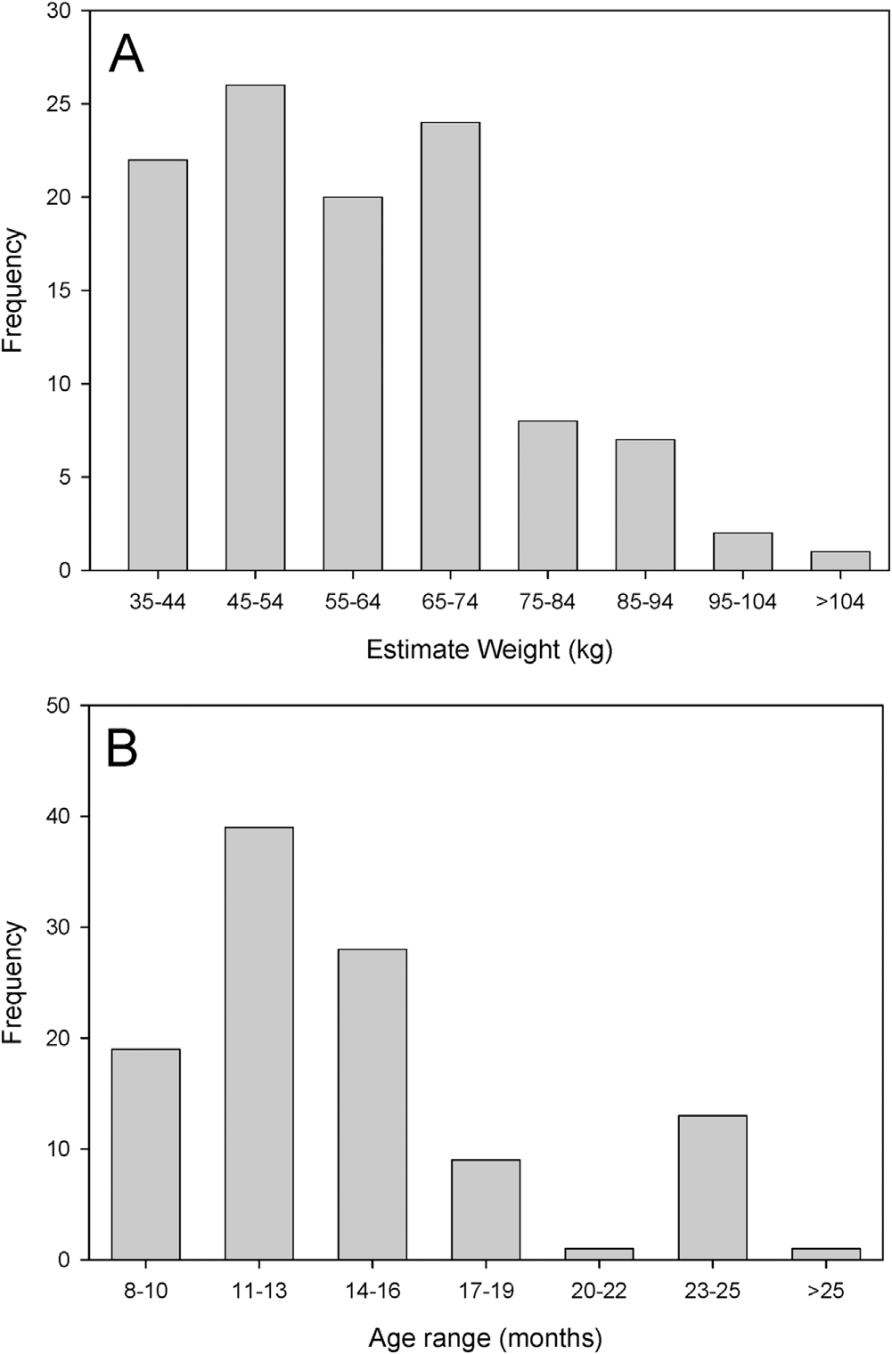
Characteristics of pigs necropsied for *Taenia solium* infection. Weight (A) and age (B) distributions of pigs from the Banke Region, Nepal, which underwent detailed necropsy examination for *Taenia solium* infection.

Animals selected for slaughter ranged between 35 and 107 kg in weight and 8–25 months of age (Fig. 1A and B). Necropsy findings are detailed in Table 1, Table 2. Thirty two of the 110 animals that were examined were found to harbour *T. solium* cysticerci (29%), of which 30 (27%) were found to have viable cysticerci (93% of the infected animals). Four animals were found to have lesions but were not recorded as being confirmed cases of *T. solium* infection; three were recorded to have a single non-viable lesion in the muscle tissue and one was found to have two viable lesions in the liver only.

**Table 1:** Numbers of *Taenia solium* cysticerci in individual, infected pigs among 110 animals subjected to post mortem examination and slicing of selected carcase sites, including all the carcase skeletal musculature from the right hand side of the carcase. Animals were 8–25 (mean 14.5) months of age from the Nepaljung region of Nepal. Cysts were detected by slicing predilection sites and half the remaining body skeletal musculature. Numbers of viable cysts are shown for various carcase locations.

Animal ID	Age (months)	Estimated total cysts (full carcass V+ NV)	Viable cysts (% all sites)	Cysts detected at necropsy (specified sites)							
				Tongue	Masseter	Diaphragm	Heart	Liver	Brain	Right forelimb	Remaining half carcass
U-20	10	3245	90.0	33	212	17	4	1	2	518	970
U-24	18	1318	99.5	0	30	27	0	0	17	253	369
U-13	12	1235	100.0	7	85	5	1	0	41	171	377
U-71	18	737	100.0	11	76	2	1	1	0	97	226
B-77	13	647	10.7	0	18	0	0	2	5	157	154
B-80	16	500	96.6	2	13	6	0	1	0	136	103
U-9	15	430	100.0	2	6	0	0	0	0	94	117
H-72	15	49	100.0	2	6	0	0	0	1	9	11
U-14	25	45	0.0	1	0	0	0	28	0	4	4
H-39	12	31	100.0	0	0	1	0	0	0	8	7
B-89	12	21	23.8	0	0	0	0	1	0	4	6
H-110	12	17	76.5	1	3	0	0	0	1	4	2
H-57	8	16	100.0	0	0	0	0	0	0	4	4
U-8	15	13	100.0	1	0	0	0	0	0	3	3
U-11	12	12	100.0	0	0	0	0	0	0	1	5
U-65	20	11	90.9	0	0	0	0	1	0	0	5
H-37	11	11	100.0	0	0	1	0	0	0	4	1
B-86	12	9	0.0	0	5	0	0	0	0	0	2
U-12	15	6	100.0	0	0	0	0	0	0	2	1
U-15	8	4	100.0	0	0	0	0	0	0	0	2
U-67	24	4	100.0	0	0	0	0	0	0	1	1
B-87	12	4	100.0	0	0	0	0	0	0	0	2
U-70	18	3	100.0	0	0	0	0	2	1	0	0
U-99	15	3	100.0	0	1	0	0	0	0	0	1
B-78	24	3	33.3	0	1	0	0	0	0	0	1
U-34	12	2	100.0	0	2	0	0	0	0	0	0
U-66	24	2	100.0	0	0	0	1	0	1	0	0
U-4	12	2	100.0	0	0	0	0	0	0	0	1
U-30	11	2	100.0	0	0	0	0	0	0	0	1
U-64	15	2	100.0	0	0	0	0	0	0	1	0
H-49	10	2	100.0	0	0	0	0	0	0	0	1
B-85	12	2	100.0	0	0	0	0	0	0	0	1

Animal ID: first letter corresponds to source (Udaypur, Hirminiya or Betahani); V: viable; NV: non-viable.

**Table 2:** Intensity of *T. solium* infection in the muscle tissues of individual pigs from the Banke Region, Nepal.

Intensity of infection (number of cysticerci in muscle tissues)	Number of animals	Proportion (%)
1–10	15	49
11–50	9	29
51–200	0	0
201–500	2	6
501–2000	4	13
2001–5000	1	3
>5000	0	0

The prevalences of infection in the three VDC were 35% in Udaipur (19/55 animals), 19% in Hirmaniya (6/31 animals) and 29% in Betahani (7/24 animals). The differences between the VDC were not statistically significantly different (Fisher’s Exact test p > 0.05).

**Fig. 2:**
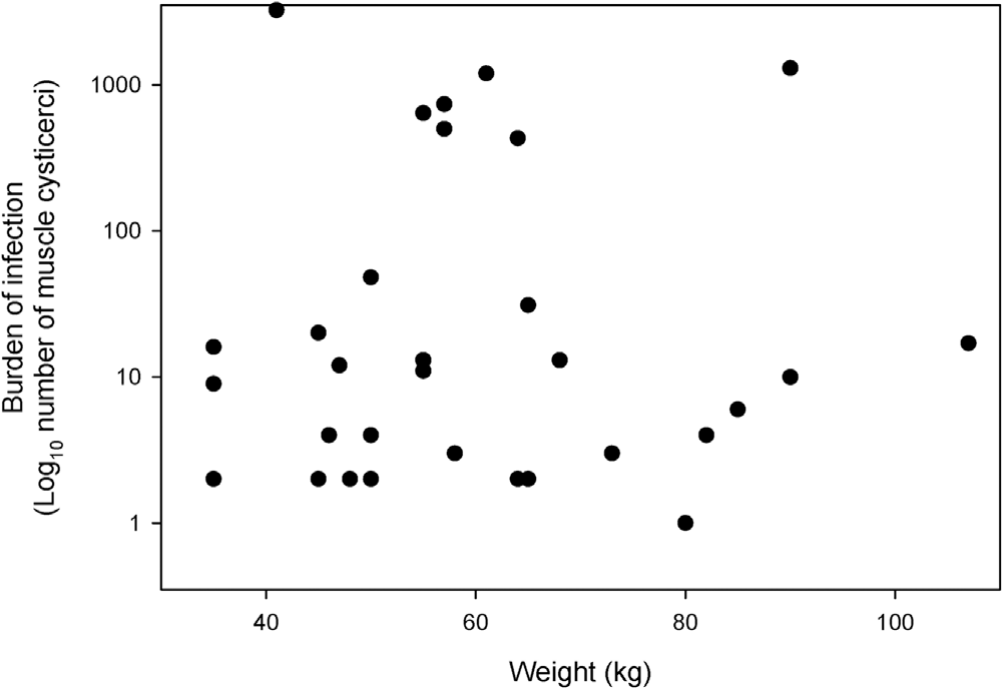
Pig age vs burden of *Taenia solium* infection relationship. Relationship between the burden of *T. solium* infection in the muscle tissues of individual pigs from the Banke Region, Nepal and the estimated body weight of the animals.

There was no significant relationship between the burden of *T. solium* cysticerci in the muscle tissues and the weight of each animal (r = −0.07558, p = 0.68; Pearson correlation coefficient; Fig. 2) or the age of each animal (r = −0.11637, p = 0.53; Pearson correlation coefficient)

## 4 Discussion

The Banke Region in Nepal was found to have one of the highest prevalences of *T. solium* infection in pigs of any region of the world where prevalence has been determined by detailed necropsy. More than a quarter of the slaughter age pigs (27%) which were necropsied were found to harbour viable *T. solium* cysts in their muscles. The great majority of the animals were reared for sale to the regional centre, Nepalgunj, a city of approximately 130,000 inhabitants. These animals present a serious risk for transmission of *T. solium* to the local communities and clearly reflect a significant level of taeniasis, and very likely neurocysticercosis, in the villages from which the animals were derived.

The survey conducted among the households from which pigs were purchased for necropsy identified many risk factors for transmission of *T. solium*, including a lack of availability or use of latrines, free-ranging pigs and little knowledge about the relationship between personal hygiene, *T. solium* infections in pigs or the symptoms of neurocysticercosis in humans. The Khatik community are predominantly farmers who rely heavily on keeping pigs as a source of food and income. The majority of these communities do not use latrines. Other communities similar to the Khatik, are present throughout the Southern parts of Nepal and the bordering area of Bihar,Uttar Pradesh, India. Traditionally, they keep indigenous breed of pig and feed mostly through scavenging. Pigs are slaughtered and consumed locally as well as sold to enter the formal meat market. Dom, Dushadh, and Mushahar are communities known to practice similar pig rearing practices like the Khatik and are found from east to west southern part of Nepal as well as in Uttar Pradesh and Bihar.

In determining the prevalence of porcine cysticercosis in this study, the assessment was made on the basis of detailed post mortem examination. Five similar assessments have been published of the prevalence of porcine cysticercosis in randomly selected, naturally exposed pigs. The highest prevalence of porcine cysticercosis was recorded in Zambia where 31 of 61 (47.7%) pigs were found to have cysticercosis (Dorny et al., 2004). In Mexico 19 of 120 animals (15.8%) were identified with *T. solium* cysticerci (Huerta et al., 2001), while in Cameroon 20 of 102 animals were found to be infected (19.6%) (Assana et al., 2010) and in Peru 18 of 107 pigs (16.8%) (Jayashi et al., 2012) and 18 of 326 pigs (5.5%) were found infected (Garcia et al., 2016). These data, which ware based on detailed necropsy examinations, provide reliable values on the prevalence of porcine cysticercosis. Recent evidence has cast doubt on the reliability of reports describing a high prevalence of porcine cysticercosis where the reports had been based on use of serological methods (Lightowlers et al., 2016). For example, in an extensive study conducted in northern Peru, 55.2% of 326 pigs were found to be positive by serology whereas only 5.5% were confirmed as being infected when the entire carcase musculature, liver and brain were sliced to reveal cysticerci (Garcia et al., 2016). Other studies have found a high rate of positive reactions in serological tests for porcine cysticercosis with sera from uninfected pigs (Devleesschauwer et al., 2013a, Gavidia et al., 2013, Jayashi et al., 2014).

Amongst the pigs that were examined from the Banke region, four animals in which lesions were identified were not included among those that were considered to have *T. solium* infection. These included three animals in which a single non-viable lesion was identified in the tissues examined. The study did not include histological or molecular analyses that may have been able to confirm the nature of these lesions. Little attention has been paid to the nature of rare fibrotic or calcified lesions in the muscle tissues of pigs, however in cattle, muscle lesions with causes other than cestode cysticerci have been described (Bockeler and Friedland, 1990, Gracey and Collins, 1992, Ogunremi et al., 2004, van der Logt and Gottstein, 2000). A similar situation is likely to be the case in pigs and, for this reason, animals detected with only a single non-viable lesion in the muscle tissues were not classified as having been infected with *T. solium*. Two other animals were found with only non-viable lesions, one with 9 lesions and one with 17 lesions. Given the number of lesions and the low likelihood that these could have any other cause than *T. solium*, these animals were included among those considered to have been infected with *T. solium*. One animal was recorded as having two viable cysticerci in the liver only. *T. hydatigena, T. saginata* and *T. asiatica* and *T. solium* are all known to have the capacity to encyst in the liver of pigs (discussed in Lightowlers et al., 2016). Testing to confirm the identity of liver lesions was not undertaken in this study, hence cysticerci in the liver were not recorded as being *T. solium* and the one individual animal having only cysticerci in the liver was not recorded as having *T. solium* infection.

The prevalence of porcine cysticercosis in the animals that were investigated in this study is likely to have been underestimated because not all the carcase skeletal musculature was sliced, due to limitations in resources that were available to undertake the post mortem studies. Vargas Mendez et al. (1986) found no difference in the distribution of *T. solium* cysts between the left and right sides of a pig carcase, hence some of the pigs from the Banke region that were found to have no cysts may have had light infections which, by probability, were present only in the carcase musculature which was not examined.

The prevalence of porcine cysticercosis described here in pigs from the Banke District of Nepal indicates that there is a high rate of *T. solium* transmission in the region and, very likely, a high rate of human cysticercosis. Further studies are warranted to determine the burden of human neurocysticercosis in the region as well as implementation of control measures to reduce transmission of the parasite (Lightowlers, 2013, Lightowlers and Donadeu, 2017).
